# The genome sequence of a non-capsular natural *Bacillus anthracis* strain of A.Br.001/002 canSNP group isolated from permafrost in Yakutia, Russia

**DOI:** 10.1128/mra.01105-24

**Published:** 2024-11-29

**Authors:** Yulia Goncharova, Irina Bakhteeva, Raisa Mironova, Yuriy Skryabin, Angelina Kislichkina, Aleksandra Solomentseva, Vitalii Timofeev

**Affiliations:** 1 Laboratory of anthrax microbiology, State Research Center for Applied Microbiology and Biotechnology (SRCAMB), Obolensk, Russia; Rochester Institute of Technology, Rochester, New York, USA

**Keywords:** *Bacillus anthracis*, anthrax, permafrost, draft genome, WGS

## Abstract

We present the results of the whole-genome sequencing of a *Bacillus anthracis* strain isolated from a permafrost sample collected in Yakutia, Russia. This strain was named YakM12. Phylogenetic analysis showed that YakM12 belongs to the canSNP group A.Br.001/002 and is genetically close to the Sterne vaccine strain.

## ANNOUNCEMENT

We describe a *Bacillus anthracis* strain isolated from a permafrost sample collected in 2016 in Yakutia (68.564567 N 144.769827 E), Russia. We have already isolated three *B. anthracis* strains from it (1). The remaining soil was frozen until 2024 when it was re-examined.

We plated the thawed at room temperature soil on selective media “Dry nutrient medium for the isolation of the anthrax pathogen” (State Research Center for Applied Microbiology and Biotechnology [SRCAMB], Russia). We found a new strain lacking capsule formation ability, named YakM12. MLVA analysis (2) showed its genetic difference from the previously described strains. Except for the absence of a capsule, it is a phenotypically typical *B. anthracis* strain, producing large, rough, dry, matte colonies with uneven edges on agar medium. In liquid medium, YakM12 forms a clot similar to a lump of cotton wool, the broth remains transparent. Bacterial cells are not mobile. YakM12 does not have lecithinase, phosphatase, or hemolytic activity, it is sensitive to anthrax bacteriophages Gamma A-26 and B-Fah and shows a positive result in the diffusion precipitation reaction with an anti-anthrax serum from horse blood (Oryol Biofactory, Russia). PCR analysis using the “MULTI-FLU real-time PCR kit” (SRCAMB, Russia) confirmed it as *B. anthracis*.

We performed a whole-genome sequencing of YakM12 (Table 1). We used BHI agar (SRCAMB, Russia) for cultivation (37°C, overnight). DNA was isolated using the Genomic DNA Purification Kit (Thermo Fisher Scientific, USA) according to the manufacturer’s instructions. The MGIEasy FS DNA Library Prep Set was used to prepare DNA libraries. WGS was performed on the DNBSEQ-G400 platform, using the high-throughput sequencing Set PE150 and high-throughput sequencing Set PE200 (2 × 150 bp and 2 × 200 bp) (all MGI Tech, China) according to the manufacturer’s instructions. A total of 38,674,548 reads were generated and *de novo* assembled using Unicycler v.0.4.7 (3) with default settings. The genome was annotated using the NCBI Prokaryotic Genome Annotation Pipeline v. 6.7 (https://www.ncbi.nlm.nih.gov/genome/annotation_prok/). The analysis of core SNPs was performed using Snippy v. 4.6.0 (4). A total of 420 core SNPs were found among 10 genomes. Visualization of the phylogenetic tree based on core SNPs was obtained using SplitsTree v. 4.19.2 using the neighbor-joining method (5).

**TABLE 1:** Summary of the draft whole-genome sequence of *Bacillus anthracis* YakM12 from permafrost

Strain	YakM12
NCBI accession no.	JBFNPM000000000
Genome size (bp)	5,356,182 bp
Number of contigs	43
N50 (bp)	889.7 kb
G + C content (%)	35
Genome coverage	2,568×
Genes	5,637
Protein coding	5,173
No. of raw reads	38,674,548 reads

**Fig 1:**
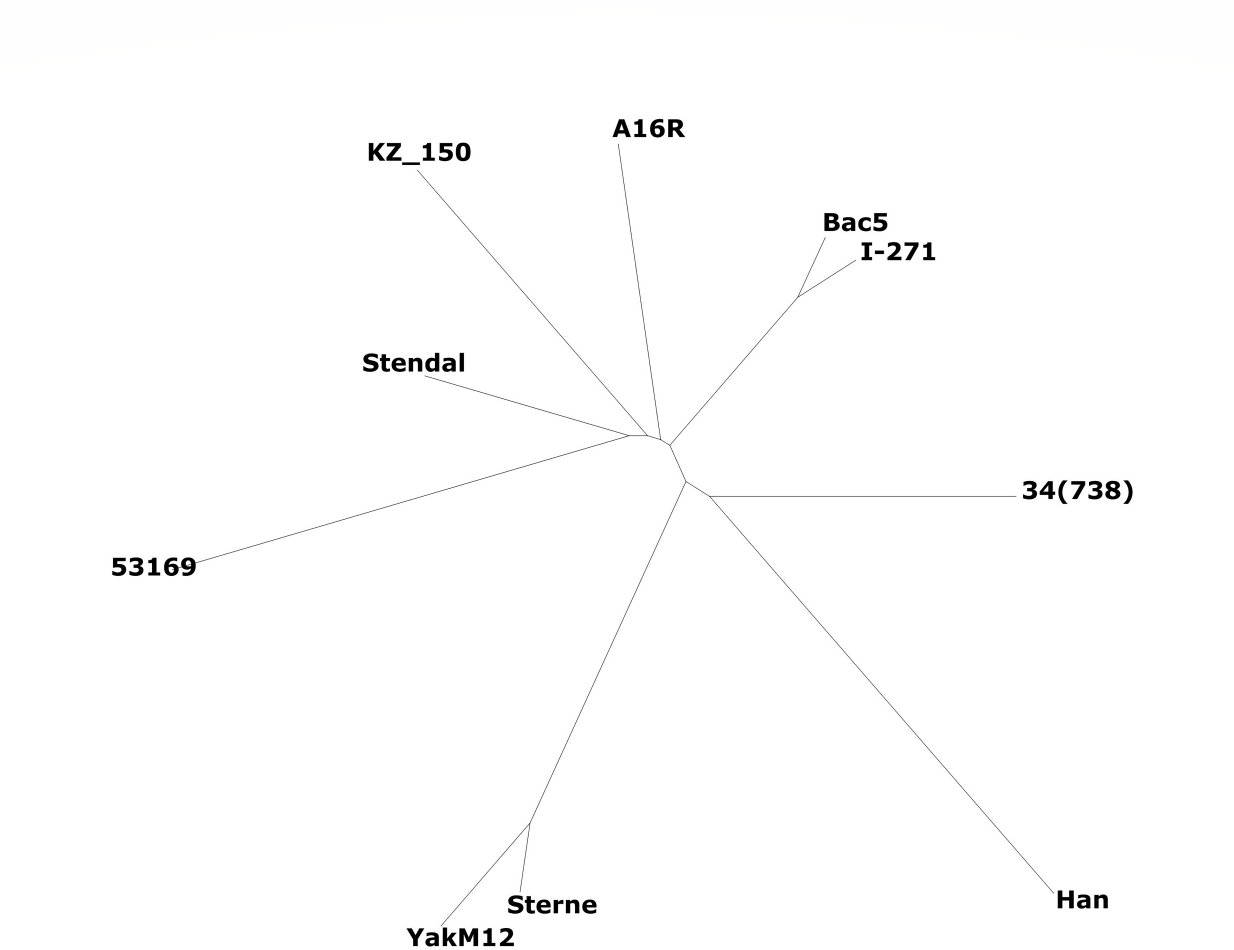
Phylogenetic relationship of the strain YakM12 with other strains of canSNP group A.Br.001/002. The following strains were used for analysis: A16R (GCA_000512775, a strain isolated in China and belongs to the L5 A16R sublinage); Sterne (GCA_000832635, a strain isolated in South Africa and belongs to the Sterne stricto sensu sublinage); Han (GCF_000747375, a strain isolated in Liaoning, China and belongs to the L6_Han sublinage); Stendal (SRR16079484, a strain isolated in Saxony-Anhalt, Germany and belongs to the L2_Stendal); KZ_150 (GCF_001543225, a strain isolated in Kazakhstan and belongs to the L4_KZ150 sublinage); 53169 (SRR25780260, a strain belongs to the L2_Stendal sublinage, isolation point is unknown); Bac5 (SRR25780262, a strain isolated in Yakutia, Russia and belongs to the L7_Bac5 sublinage); 34(738) (SRR25780266, a strain isolated in Kazakhstan and belongs to the L8_34(738) sublinage); I-271 (SRR25780267, a strain isolated in Yamal peninsula, Russia and belongs to the L7_Bac5 sublinage).

The YakM12 strain belongs to the canSNP group A.Br.001/002 (6). We recently identified two strains of this group in the Russian Arctic, on the Yamal Peninsula, and in Yakutia, which are genetically similar, forming a new, Arctic clade within the A.Br.001/002 group (7). A phylogenetic analysis of the YakM12 strain, comparing it to a set of strains of the A.Br.001/002 group, was performed (Fig. 1).

The YakM12 strain does not belong to the Arctic clade and is genetically close to the Sterne vaccine strain (29 SNPs), belonging to the Sterne stricto sensu sublineage (8). We consider laboratory contamination unlikely as all permafrost manipulations were carried out in BSL3 facilities, where work with avirulent strains such as Sterne has never been carried out.

Instead, we consider two possible scenarios. The first involves the possible import of the Sterne vaccine to the northern USSR in the early 20th century, leading to environmental exposure from a vaccinated animal in free grazing. The strain accumulated several dozen point mutations in a limited soil life cycle. An alternative, more speculative scenario assumes the introduction of a common ancestor of the Sterne and YakM12 strains from a certain habitat (e.g., China, where the A.Br.001/002 group is widespread) to geographically distant regions—Yakutia and South Africa, where they evolved independently and independently lost the pXO2 plasmid—the Sterne strain as a result of laboratory manipulations, and the YakM12 strain during the spore germination-spore formation cycles in the soil.

## Data Availability

Raw data for *Bacillus anthracis* YakM12 are deposited at NCBI through the SRA genome submission portal under the accession number SRR29660463, the genome sequences are available under accession number JBFNPM000000000. The version deposited in this paper is the first version JBFNPM000000000.1.
